# A review of the genus *Serangium* Blackburn (Coleoptera, Coccinellidae) from China

**DOI:** 10.3897/zookeys.134.1715

**Published:** 2011-10-06

**Authors:** Xing-Min Wang, Shun-Xiang Ren, Xiao-Sheng Chen

**Affiliations:** 1 Engineering Research Center of Biological Control, Ministry of Education, South China Agricultural University, Guangzhou, 510642, China; 2 Institute of Zoology, Chinese Academy of Sciences, Beijing, 100101, China

**Keywords:** Coleoptera, Coccinellidae, *Serangium*, new species, China

## Abstract

The genus *Serangium* Blackburn from China is reviewed. The genus *Catanella* Miyatake is removed from synonymy with *Serangium*. *Serangium baculum* Xiao is transferred to *Catanella*, as *Catanella baculum* (Xiao), **comb. n.** Twelve species of *Serangium* are described, keyed and illustrated, including eight new species, *Serangium magnipunctatum* Wang & Ren, **sp. n.**, *Serangium trimaculatum* Wang & Ren, **sp. n.**, *Serangium centrale* Wang & Ren, **sp. n.**, *Serangium leigongicus* Wang & Ren, **sp. n.**, *Serangium latilobum* Wang & Ren, **sp. n.**, *Serangium digitiforme* Wang & Ren, **sp. n.**, *Serangium dulongjiang* Wang, Ren & Chen, **sp. n.**, and *Serangium contortum* Wang & Ren, **sp. n.** *Serangium punctum* Miyatake is newly recorded from China.

## Introduction

The genus *Serangium* was erected by Blackburn (1889) with *Serangium mysticum* Blackburn, 1889, from Australia as the type species. Chapin (1940) pointed out that *Serangium* and its allied genera form a coherent group, but he did not proposed a tribal name for this group. The name Serangiini was introduced by Blackwelder (1945) in his checklist and was validated by Pope (1962). The tribe Serangiini is now classified in the subfamily Microweiseinae (Ślipiński, 2007).

*Serangium* is the largest genus of Serangiini with 45 described species, mostly occurring in the Oriental Region (Miyatake 1994; Ślipiński and Burckhardt 2006), with 4 species known from China (Sasaji 1967; Pang and Mao 1979; Xiao and Li 1992; Pang et al. 2004; Ren et al. 2009). Recent collecting in the southern half of China has revealed several more species, which are described here. 12 species of *Serangium* from China are reviewed, described and illustrated, of which eight are new.

## Materials and methods

The specimens examined were collected from China. All collected species were preserved in 85% ethanol. External morphology was observed with a dissecting stereoscope (SteREO Discovery V20, Zeiss). The following measurements were made with an ocular micrometer: total length, length from apical margin of clypeus to apex of elytra (TL); Total width, width across both elytra at widest part (TW=EW); height, take from the highest part of the beetle (TH); head width (HW); pronotal length, from the middle of anterior margin to the base of pronotum (PL); pronotal width at widest part (PW); elytral length, along the suture, from the apex to the base including the scutellum (EL). Male and female genitalia were dissected, cleared in 10% solution of NaOH by boiling for several minutes, and examined with an Olympus BX51 compound microscope.

Images were photographed with digital cameras (AxioCam HRc and Coolsnap–Pro*cf* & CRI Micro*Color), connected to the dissecting microscope. The software AxioVision Rel. 4.8 and Image–Pro Plus 5.1 were used to capture images from both cameras, and photos were cleaned up and laid out in plates with Adobe Photoshop CS 8.0.

Terminology follows Ślipiński (2007). Type specimens depositions are indicated by SCAU for South China Agriculture University, Guangzhou; and IOZ for the Institute of Zoology, Institute of Zoology, Chinese Academy of Sciences, Beijing. Other examined type specimens are from Kunming Institute of Zoology, Chinese Academy of Sciences (KIZ).

## Taxonomy

### Genus Serangium

Blackburn, 1889

http://species-id.net/wiki/Serangium

Serangium  Blackburn, 1889: 187, 209. Type species, monotypy: *Serangium mysticum*Blackburn, 1889.
Serangium : Sicard, 1909: 150, 151; Chapin, 1940: 268; Miyatake, 1961b: 50; Sasaji, 1971: 52; Pang & Mao, 1979: 27; Miyatake, 1994: 238; Ślipiński & Burckhardt, 2006: 39; Ślipiński, 2007: 53.
Semichnoodes  Weise, 1892: 15. Type species, monotypy: *Semichnoodes kunowi* Weise, 1892. Synonymized by Weise, 1908: 13.
Catana  Chapin, 1940: 266. Type species, original designation, *Catana clauseni* Chapin, 1940. Synonymized by Ślipiński & Burckhardt, 2006: 39.

#### Diagnostic description.

**Figures 1–12.**
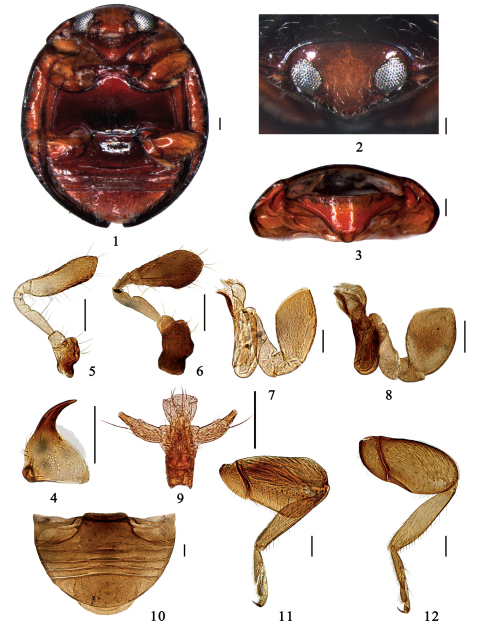
**1–5, 7, 9–12.** *Serangium japonicum* Chapin. **1** ventral view **2** frontal view **3** prothorax **4** mandible **5** antenna **7** maxilla **9** labium **10** abdomen **11** front leg **12** hind leg; **6, 8** *Serangium centrale*Wang & Ren, sp. n. **6** antenna **8** maxilla. Scale bars: 0.1mm.

**Figures 13–18.**
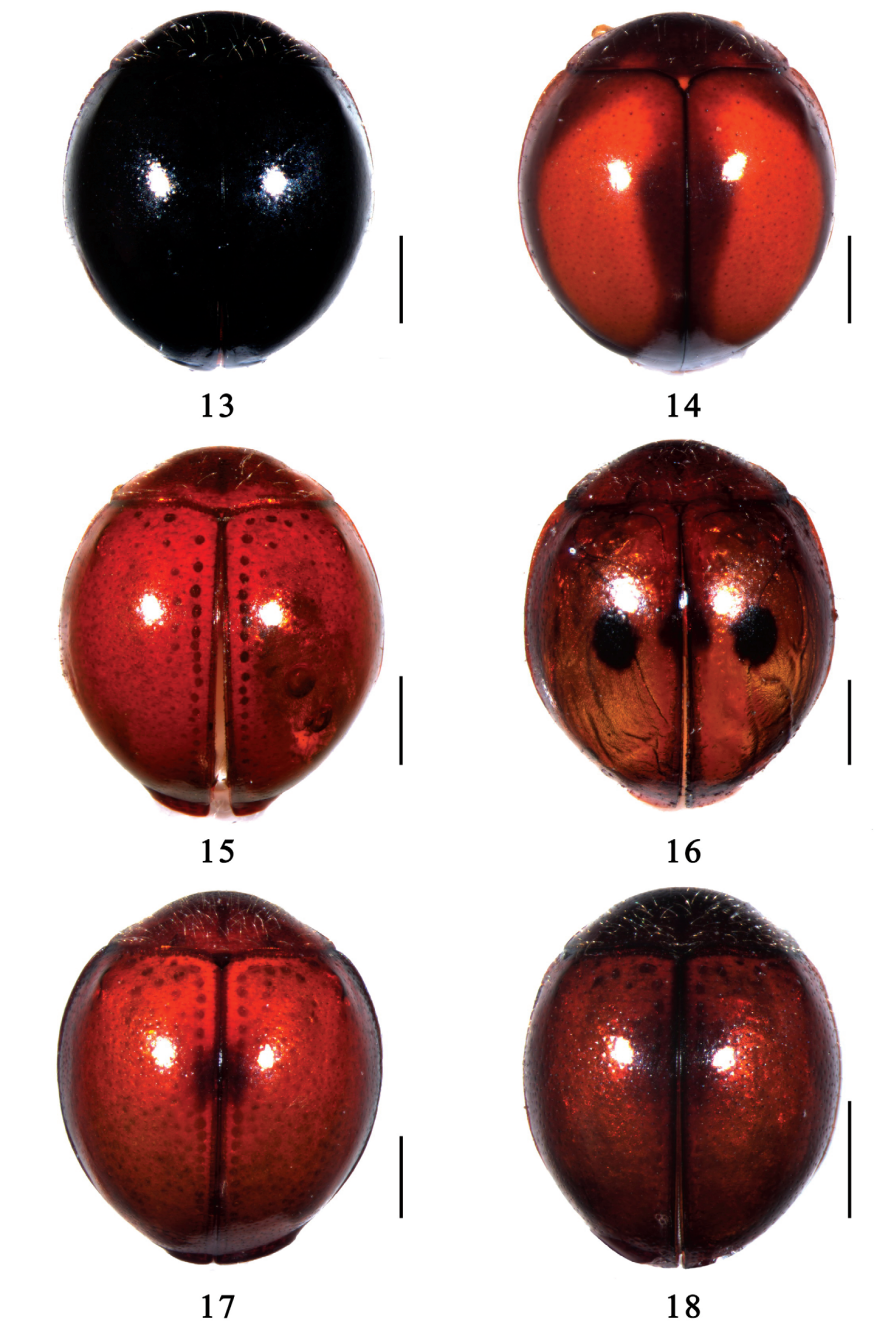
Dorsal view.**13** *Serangium japonicum* Chapin **14** *Serangium clauseni*(Chapin) **15** *Serangium magnipunctatum*Wang & Ren, sp. n. **16** *Serangium trimaculatum*Wang & Ren, sp. n. **17** *Serangium centrale*Wang & Ren, sp. n. **18** *Serangium leigongicus* Wang & Ren, sp. n. Scale bars: 0.5mm.

Body minute, hemispherical with head in repose drawn into prothorax and closely fitting ventrally against prominent prosternal lobe (Fig. 1); dorsum glabrous, except pronotum with sparse setae, and sometimes elytral margins (Fig. 13). Head transverse, ventrally flattened with clypeal region prominent anteriorly (Fig. 2); frontoclypeus deeply emarginated around exposed antennal insertions. Mandible normal, with single apical tooth (Fig. 4); terminal maxillary palpomere always longer than wide, barrel shaped, truncate at apex (Figs 7–8). Antenna 9-segmented, antennomere 3 moderately to strongly elongate, terminal segment forming a large club which is always spatulately elongate or elongate oval and flat, apex angular (Figs 5–6).

Pronotum short, strongly transverse. Scutellum relatively large, triangular. Elytra strongly convex, usually smooth without visible punctures. Winged; wings with greatly reduced venation. Prosternum strongly prominent medially forming a broad lobe concealing mouthparts from below; prosternal process subtruncate apically, broad (Fig. 3). Epipleuron moderately narrow, incomplete, reaching 2/3 of elytra length, with clearly delimited cavities to accommodate apices of mid and hind femora. Abdomen with 5 ventrites (Fig. 10). Postcoxal line at abdominal ventrite 1 incomplete, reaching lateral margin of the ventrite, without associated pits or pores. Femora, especially profemur, broad, flat closely fitting into depressions on ventral surface protecting tibiae and tarsi from below; tarsus 4-segmented (Figs 11–12).

Male genitalia: tegmen strongly asymmetrical, parameres short or distinctly reduced with sparse setae apically (Figs 27–28). Female genitalia: ovipositor triangularly elongate, weakly sclerotised usually bearing short styli; infundibulum absent, spermatheca small and well sclerotised (Fig. 29).

#### Remark:

*Catana* Chapin, 1940 and *Catanella* Miyatake, 1961a were synonymised with *Serangium* by Ślipiński and Burckhardt (2006) in review of the Australian Serangiini. While working on the Chinese species of Serangiini, we have examined specimens of *Catana clauseni*Chapin in China, and agreed with Ślipiński & Burckhardt’s opinion that *Catana* is synonymous with *Serangium*. We have also examined many specimens of *Catanella formosana* Miyatake, 1961a and found that *Catanella*’s 8-segmented antenna and 3-segmented tarsus are constant, while male and female genitalia are also different from *Serangium*. Thus, *Catanella* should be a valid genus.

Pang et al. (2004) recorded *Serangium lygacum* Iablokoff-Khnzorian, 1972 from China. After our re-examination of this specimen, we conclude that it was an incorrect identification of *Serangium drepnicum* Xiao, 1992.

In addition, Xiao and Li (1992) described a new species *Serangium baculum* with 9-segmented antenna and 4-segmented tarsus. We have examined type materials of *Serangium baculum* and found that its antenna has 8-segments and tarsus is 3-segmented which is similar to the characters of the genus *Catanella*. Therefore we consider that *Serangium baculum* is wrongly placed in *Serangium* and should be moved to *Catanella* (*Catanella baculum* (Xiao) comb. n.)

#### Key to species of Serangium from the China

**Table:** 

1	Terminal antennal segment spatulately elongate	1
–	Terminal antennal segment elongate oval and flat, apex angular	4
2	Dorsum uniformly dark, without spots (Fig. 13). TL: 1.60–2.08mm, TW: 1.40–1.98mm	*Serangium japonicum* Chapin
–	Dorsum dark brown to black, with spots, or dorsum red without spots	3
3	Elytra reddish to dark brown, with a large scarlet reniform spot on each elytron (Fig. 14). Paramere 2/3 of penis guide length (Fig. 32). TL: 1.95–2.24mm, TW: 1.66–1.98mm	*Serangium clauseni* (Chapin)
–	Elytra red, or red with wide black margin (Fig. 15). Paramere 2/3 of penis guide length (Fig. 38). TL: 2.09–2.18mm, TW: 1.75–1.85mm	*Serangium magnipunctatum* sp. n.
4	Dorsum orange to burgundy, with at least one dark spot on suture	5
–	Dorsum uniform dark, without spots	7
5	Dorsum reddish brown to burgundy, without black spots on elytral disc	6
–	Dorsum orange with black spots on each elytron (Fig. 16). TL: 2.09–2.21mm, TW: 1.75–1.88mm	*Serangium trimaculatum* sp. n.
6	Pronotum and elytra reddish brown, with a dark spot in the middle of elytral suture (Fig. 17). TL: 2.08–2.27mm, TW: 1.88–1.98mm	*Serangium centrale* sp. n.
–	Pronotum distinctly darker than elytra, elytra burgundy without spot (Fig. 18). TL: 1.58mm, TW: 1.35mm	*Serangium leigongicus* sp. n.
7	Metaventrite basal half with distinct median discrimen	8
–	Metaventrite basal half without median discrimen	10
8	Punctures around median discrimen of metaventrite very large and dense, with short thick setae	9
–	Punctures around median discrimen of metaventrite fine and sparse, with short sparse setae. Penis guide flat and elongated tongue-shaped (Fig. 71). TL: 1.98–2.11mm, TW: 1.68–1.81mm	*Serangium drepnicum* Xiao
9	Penis has a small pominence at 1/5 length (Fig. 57). Penis guide wide at basal half, sharply narrowed in middle, with a finger-shape apex (Fig. 59). TL: 2.14–2.18mm, TW: 1.91–1.96mm	*Serangium latilobum* sp. n.
–	Penis distinctly broadening at apical 1/5–2/5 length (Fig. 63). penis guide widest at base, gradually narrowing to middle, with a finger-shape apex (Fig. 65). TL: 2.14–2.21mm, TW: 1.80–1.85mm	*Serangium digitiforme* sp. n.
10	Apex of penis rounded. Tegmen asymmetrical but simple	11
–	Apex of penis sharply pointed (Fig. 75). Tegmen asymmetrical and very complex (Fig. 77). Punctures on frons very large and dense. TL: 2.08mm, TW: 1.85mm	*Serangium punctum* Miyatake
11	Penis is long and slender (Fig. 81). Penis guide is elongated tongue-shaped (Fig. 83). The first part of spermatheca has a strong constriction in middle (Fig. 84). TL: 2.01–2.14mm, TW: 1.68–1.81mm	*Serangium dulongjiang* sp. n.
–	Penis is moderately long and stout (Fig. 87). Penis guide is contorted (Fig. 89). The first part of spermatheca has a feebly constriction in middle (Fig. 90). TL: 1.62–1.78mm, TW: 1.42–1.58mm	*Serangium contortum* sp. n.

### Serangium japonicum

Chapin, 1940

http://species-id.net/wiki/Serangium_japonicum

Figs 1–5, 7, 9 13 25–29 92

Serangium japonicum Chapin, 1940: 269; Miyatake, 1961b: 140–142; Sasaji, 1971: 55; Pang & Mao, 1979: 28; Cao, 1992: 106; Ślipiński & Burckhardt, 2006: 50; Ren et al., 2009: 40.

#### Diagnosis.

This species can be identified by its completely black body with yellowish brown anterior angles of pronotum, and spatulately elongate terminal antenna segment, flattened and elongate tongue-shape penis guide in ventral view. (Fig. 28).

#### Description.

 TL: 1.60–2.08mm, TW: 1.40–1.98mm, TH: 0.82–1.02mm, TL/TW: 1.14–1.15; PL/PW: 0.52–0.53; EL/EW: 0.94–0.98.

Body minute, hemispherical, dorsum strongly convex, shiny and glabrous (Fig. 13). Dorsum uniformly black, except anterior angles of pronotum yellowish brown. Head yellowish brown. Underside reddish brown, except metaventrite dark brown. Legs yellowish brown (Fig. 1).

Head transverse and ventrally flattened, 0.40× elytral width (HW/EW=1: 2.50); punctures on frons fine and sparse, separated by 1.5–6.0 times their diameter, with short sparse setae (Fig. 2); eyes moderately large and coarsely faceted, widest interocular distance 0.45× width of head (Fig. 2). Antenna 9-segmented, terminal segment large, spatulately elongate (Fig. 5).

Pronotum short and strongly transverse, 0.58× elytral width (PW/EW=1: 1.72), densely covered in fine punctures associated with long sparse setae, punctures slightly larger than those on head, separated by 1.0–3.0 times their diameter. Punctures on elytra fine and sparse, similar to those on pronotum, with a few long setae at humeral angles and a row of evenly spaced setae along margin. Prosternum shiny, glabrous and impunctate with sparse setae. Mesoventrite small, transverse, surface mat weakly furrowed, sparsely setae. Metaventrite shiny, basal half with distinctly median discrimen; punctures around median discrimen very large and dense, with short thick setae, and on other parts fine and sparse, separated by 2.0–6.0 times their diameter, with short sparse setae.

Male genitalia. Penis strongly curved in whole length, apex shortly narrowing and rounded, penis capsule indistinct (Fig. 26). Tegmen rather slender and strongly asymmetrical (Figs 27–28). Penis guide in ventral view flattened and elongate tongue-shape. Left paramere in ventral view flat and short bearing a few long setae, and right piece short but distinctly projecting, bearing a few long setae (Fig. 28). Penis guide in lateral view long and thin, almost straight, apex pointed. Right paramere in lateral view about 1/2 of penis guide (Fig. 27).

Female genitalia. Genital plate elongate triangular with a rounded apex, sparsely hairy on the apical portion, stylus rather long, bearing few setae (Fig. 29). Spermatheca divided into two parts, one of which is somewhat globular with a feeble constriction and two small pinch-like projections, the other is tubular, becoming slightly more slender distally (Fig. 29).

#### Specimens examined.

 **China, Anhui:** 4♂♂1♀, Guniujiang National Natural Reserve, Shitai, 29°58.97'N, 117°39.51'E , ca 490m, 24.ix.2010, Wang XM et al. leg.; **Fujian:** 6♂♂6♀♀, Fuzhou, 26°4.66'N, 119°22.05'E , ca 30m, 15.vi.1983, Tang YQ leg.; 1♂3♀♀, Shaowu, 27°20.56'N, 117°28.75'E , ca 220m, 24.viii.1984, Pang XF leg.; **Guangdong:** 4♂♂4♀♀, Gaoyao, 23°01.97'N, 112°26.41'E , ca 40m, 4.xii. 1985, Jiang N. leg.; 1♂, Shipai Country, Guangzhou, 23°7.85'N, 113°20.49'E , ca 5m, 29.vii.29, Ren SX leg.; 2♂♂2♀♀, Chenjia, Yangshan, 24°45.51'N, 112°51.68'E , ca 310m, 9.vii. 1996, Tian MY leg.; 4♂♂5♀♀, Shimentai Mountain, Yingde, 24°23.83'N, 113°17.24'E , ca 550m, 3.xi.2004, Wang XM et al. leg.; 2♂♂, campus of SCAU, 23°9.49'N, 113°21.07'E , ca 30m, viii.2008, Wang XM leg.; 1♂, Qinshuigu, Nanling Mountain, Shaoguan, 24°54.95'N, 113°6.46'E , ca 560m, 29. ix.2004, Wang XM leg.; **Guangxi:** 9♂♂10♀♀, Guilin, 25°13.93'N, 110°15.20'E , ca 300m, 23.ix.1987, Pang XF leg.; 1♀, Hongqilinchang, Shiwandashan Mountian, Shangsi, 21°54.45'N, 107°54.57'E , ca 350m, 10. xi. 2004, Lv XB leg.; 1♂, Maoershan Mountain, Guilin, 25°49.51'N, 111°01.07'E , ca 440m, 16.x. 2004, Wang XM leg.; **Guizhou:** 1♀, Huaxi, Guiyang, 26°24.60'N, 106°40.07'E , ca 1120m, 11.viii.1987, Peng XF leg.; 12♂♂17♀♀, Suoluo National Natural Preserve, Chishui, 28°26.42'N, 105°59.80'E , ca 430m, 9.viii. 1994, Tian MY leg.; 1♂1♀, Xifeng, 27°5.35'N, 106°44.59'E , ca 1100m, 9.viii.1997. Peng ZQ leg.; 2♂♂3♀♀, Dongtang, Libo, 25°17.28'N, 108°0.94'E , ca 730m, 15.x.2008, Liang JB leg.; 2♂♂, Leigongshan Moutain, Leishan, 26°22.71'N, 108°11.03'E , ca 1500m, 30.vii.1997, Peng ZQ leg.; 1♂3♀♀, Sanchahe, Libo, 25°24.53'N, 108°5.22'E , ca 730m, 19.x.2008, Liang JB leg.; **Hainan:** 2♂♂1♀, Jianfengling, 18°44.49'N, 108°51.85'E , ca 840m, 3.xi.1989, Ren SX leg.; 2♂♂, Jianfengling, 18°44.49'N, 108°51.85'E , ca 840m, ix.1995, Peng ZQ leg.; **Hubei:** 1♂1♀, Wudangshan Mountain, Shiyan, 32°24.50'N, 111°1.31'E , ca 1090m, 17.vii.1997, Peng ZQ leg.; **Hunan:** 1♂, Zhubotang, Yiyang, 28°29.07'N, 112°26.70'E , ca 50m, 18.viii.2001, Peng ZQ leg.; **Sichuan:** 2♂♂3♀♀, Zhongxian, 30°18.78'N, 107°59.80'E , ca 360m, 24.viii.1989, Ren SX leg.; 1♂1♀, Kaixian, 31°9.55'N, 108°24.26'E , ca 330m, 25.vi.1989, Ren SX leg.; 3♂♂4♀♀, Jiulidi, 30°42.80'N, 104°3.16'E , ca 500m, 27.vi.1983, Pang XF leg.; 4♂♂4♀♀, Puge, 27°35.32'N, 102°25.98'E , ca 2000m, 15.ix.2007, Wang XM leg.; 10♂♂16♀♀, Pangzhihua, 26°36.69'N, 101°35.35'E , ca 1200m, 1400m, 16.ix.2007, Wang XM leg.; 1♂1♀, Miyi, 26°50.01'N, 102°3.86'E , ca 1150m, 30.ix.2000, Peng ZQ leg.; **Zhejiang:** 4♂♂6♀♀, Cixi, 30°8.72'N, 121°18.91'E , ca 70m, 18.x.1988, Yu GY leg.; **Yunnan:** 2♂♂2♀♀, Ailaoshan, Mountain, Jingdong, 24°25.41'N, 101°3.80'E , ca 2000m, 1.x.2006, Wang XM et al. leg.; 1♂, Yingjiang, 24°36.53'N, 97°43.95'E , ca 1500m, 20.ix.2006, Wang XM leg.

#### Distribution.

China (Anhui, Chongqing, Fujian, Guangdong, Guangxi, Guizhou, Hainan, Hubei, Hunan, Shanghai, Shaanxi, Sichuan, Taiwan, Zhejiang, Yunnan); Japan.

### Serangium clauseni

(Chapin, 1940)

http://species-id.net/wiki/Serangium_clauseni

Figs 14 30–35 92

Catana clauseni Chapin, 1940: 267; Miyatake, 1961b: 139; Ren et al., 2009: 36.
Serangium clauseni : Ślipiński & Burckhardt, 2006: 50.

#### Diagnosis.

This species can be identified by the unique dorsal color pattern and spatulately elongate terminal antenna segment (Fig. 14). The male genitalia are similar to *Serangium japonicum*, but it can be distinguished from latter as follow: penis is stout (Fig. 31), penid guide in ventral view wider than latter (Fig. 33) and right paramere in lateral view is stout and 2/3 of penid guide (Fig. 32).

#### Description.

 TL: 1.95–2.24mm, TW: 1.66–1.98mm, TH: 0.89–1.15mm, TL/TW: 1.13–1.17; PL/PW: 0.44–0.47; EL/EW: 0.93–0.95.

Body minute, hemispherical, dorsum strongly convex, shiny and glabrous (Fig. 14). Head orange to brown, pronotum reddish to dark brown, except light-colored anterior angles. Scutellum reddish to dark brown and ground color of elytra reddish to dark brown, with a large reniform spot on each elytron bright castaneous. Underside yellowish to reddish brown, except prosternum dark brown. Legs yellowish brown.

Head transverse and ventrally flattened, 0.41× elytral width (HW/EW=1: 2.45); punctures on frons moderated large, separated by 1.5–4.0 times their diameter, with short sparse setae; eyes moderately large and coarsely faceted, widest interocular distance 0.37× head width. Antenna 9-segmented, terminal segment large, spatulately elongate.

Pronotum short and strongly transverse, 0.68× elytral width (PW/EW=1: 1.46), sparsely covered in moderated large punctures associated with long sparse setae, punctures similar to those on head, separated by 2.0–6.0 times their diameter. Punctures on elytra very fine and sparse, smaller than those on pronotum, separated by 3.0–8.0 times their diameter, with a row of evenly spaced setae along margin. Prosternum shiny and glabrous, with sparse punctures and setae. Mesoventrite small, transverse, surface mat, weakly furrowed. Metaventrite shiny, basal half with distinctly median discrimen; punctures around median discrimen very large and dense, with short thick setae, and on other parts moderated large, sparse, separated by 2.0–5.0 times their diameter, with short sparse setae.

Male genitalia. Penis stout, strongly curved in whole length, apex narrowing and rounded, penis capsule indistinctly (Fig. 31). Tegmen slender and strongly asymmetrical (Figs 32–33). Penis guide in ventral view flattened and tongue-shape, slightly wider and shorter than *Serangium japonicum* (Fig. 33). Left paramere in ventral view flat and short bearing a few long setae, and right piece short but distinctly projecting, bearing a few long setae. Penis guide in lateral view thin and straight, apex pointed. Right paramere in lateral view stout, about 2/3 of penis guide (Fig. 32).

Female genitalia. Genital plate elongate triangular with a rounded apex, sparsely hairy on the apical portion, stylus rather long, bearing few setae (Fig. 35). Spermatheca divided into two parts, one of which is somewhat globular with a feeble constriction and two small pinch-like projections, the other is tubular, becoming slightly more slender distally (Fig. 34).

#### Specimens examined.

 **China, Hainan:** 9♂♂23♀♀, Wuzhishan, 18°47.07'N, 109°31.97'E , ca 700m, 3.v.1996, Peng ZQ leg.; 4♂♂3♀♀, Wushi, 19°8.99'N, 109°53.84'E , ca 320m, 14.vii.1999, Peng ZQ leg.

#### Distribution.

China (Hainan).

### Serangium magnipunctatum

Wang & Ren sp. n.

urn:lsid:zoobank.org:act:7F91655E-BBB2-4219-BAAD-7680A9E0BDE6

http://species-id.net/wiki/Serangium_magnipunctatum

Figs 15 36–41 92 

#### Diagnosis.

This species is similar to *Serangium clauseni* in general appearance and male genitalia, from which it differs in its dorsal color pattern, elytra with a few large inner punctures at basal margins and along elytral suture (Fig. 15). The male genitalia are also diagnostic: penis is slightly thinner than *Serangium clauseni* (Figs 31, 37), paramere about 1/3 of penis guide which is 2/3 in *Serangium clauseni* (Figs 32, 39).

#### Description.

 TL: 2.09–2.18mm, TW: 1.75–1.85mm, TH: 1.02–1.05mm, TL/TW: 1.18–1.20; PL/PW: 0.47–0.49; EL/EW: 0.96.

Body minute, hemispherical, dorsum strongly convex, glabrous (Fig. 15). Head yellow, pronotum reddish brown to black, except light-colored anterior angles and basal margin. Scutellum reddish brown to black. Elytra red to black, sometimes elytra black with median part red. Underside reddish brown and legs yellow.

Head transverse and ventrally flattened, 0.42× elytral width (HW/EW=1: 2.41); punctures on frons fine and conspicuous, separated by 1.5–5.0 times their diameter, with short sparse setae; eyes moderately large and coarsely faceted, widest interocular distance 0.45× head width. Antenna 9-segmented, terminal segment large, spatulately elongate.

Pronotum short and strongly transverse, 0.72× elytral width (PW/EW=1: 1.39), sparsely covered in fine punctures associated with long sparse setae, punctures smaller than those on head, separated by 2.0–4.0 times their diameter. Elytra with a few large inner punctures at basal margins and two rows of large punctures along elytral suture. Pro- and mesoventrites mat and weakly furrowed, with short sparse setae. Metaventrite shiny and glabrous, basal half with distinctly median discrimen; punctures around median discrimen large and dense, with short thick setae.

Male genitalia. Penis strongly curved in whole length, apex shortly narrowing and rounded, penis capsule indistinctly (Fig. 37). Tegmen rather slender and strongly asymmetrical (Figs 38–39). Penis guide in ventral view flattened and tongue-shape (Fig. 39). Left paramere in ventral view very short bearing a few long setae, and right piece short and stout, distinctly projecting, bearing a few long setae (Fig. 39). Penis guide in lateral view thin and straight, apex pointed. Right paramere in lateral view short, about 1/3 of penis guide (Fig. 38).

Female genitalia. Genital plate elongate triangular with a rounded apex, sparsely hairy on the apical portion, stylus long, bearing few setae (Fig. 41). Spermatheca divided into two parts, one of which is somewhat globular with a feeble constriction and two small pinch-like projections, the other is tubular, becoming slightly more slender distally (Fig. 40).

#### Type materials.

 **Holotype:** 1♂, **China, Yunnan:** Jiluoshan, Xishuangbanna, 21°58.78'N, 101°7.79'E , ca 1020m, 28.iv.2008, Wang XM leg. (SCAU). **Paratypes** (19): Guangxi: 1♂, Guilongshan, Napo, 23°21.63'N, 105°41.74'E , ca 880m, 4.viii.2005, Wang XM leg. (SCAU); **Yunnan:** 2♀♀, Mengxing, Mengla, 21°52.63'N, 101°27.07'E , ca 690m, 1000m, 13.v.2008, Wang XM leg. (SCAU); 4♂♂12♀♀, Lincang, 23°52.56'N, 100°5.88'E , ca 1460m, 27.viii.2005, Wang XM et al. leg. (2♂♂6♀♀ SCAU, 2♂♂6♀♀ IOZ).

#### Distribution.

China (Guangxi, Yunnan).

#### Etymology.

The specific epithet is formed from the Latin adjective *magnus* and *punctatus*, referring to elytra with large inside punctures.

### Serangium trimaculatum

Wang & Ren sp. n.

urn:lsid:zoobank.org:act:4E8202EC-9D22-4B90-B72C-7D66DC77897A

http://species-id.net/wiki/Serangium_trimaculatum

Figs 16 42–45 93 

#### Diagnosis.

This is a very distinctive species having three black spots on the elytra (Fig. 16). The male genitalia are similar to *Serangium punctum*, but can be distinguished from latter by long left paramere and apexof penis guide (Figs 44–45).

#### Description.

 TL: 2.09–2.21mm, TW: 1.75–1.88mm, TH: 0.79–0.89mm, TL/TW: 1.18–1.20; PL/PW: 0.43–0.47; EL/EW: 0.89–0.93.

Body minute, hemispherical, dorsum strongly convex, shiny and glabrous (Fig. 16). Head orange, pronotum and scutellum orange. Elytra orange, with three black spots: two on the center of elytra and one on the middle of suture. Underside yellowish brown, except pro-, meso- and metaventrites black. Legs yellowish brown.

Head transverse and ventrally flattened, 0.38× elytral width (HW/EW=1: 2.65); punctures on frons moderated large, separated by 0.5–1.5 times their diameter, with long sparse setae; eyes moderately large and coarsely faceted, widest interocular distance 0.58× head width. Antenna 9-segmented, terminal segment large, elongate oval and flat, apex angular.

Pronotum short and strongly transverse, 0.70× elytral width (PW/EW=1: 1.43), sparsely covered in fine punctures associated with long sparse setae, punctures smaller than those on head, separated by 1.5–4.0 times their diameter. Punctures on elytra very fine and sparse, similar to those on pronotum, with a row of evenly spaced setae along margin. Prosternum glabrous, punctures fine and sparse, separated by 2.0–3.0 times their diameter, with a few short setae. Mesoventrite small, transverse, surface shiny and impunctate. Metaventrite shiny, basal half with distinctly median discrimen; punctures inconspicuous, with short sparse setae.

Male genitalia. Penis long and slender, strongly curved in whole length, apical half of siphon strongly narrowing apical, and apex very thin and sharply pointed, penis capsule broadening basally and with a distinct inner process (Fig. 43). Tegmen strongly asymmetrical and extremely complicate (Figs 44–45). Penis guide moderatedly elongate, with a finger-like apex, a small prominence at right side near the apex, bearing many distinct hairs dorsally (Fig. 45). Right lateral lobe of tegmen short, bearing dense setae. Left lateral lobe of tegmen relatively long, rounded apical without any setae (Fig. 45). Basal piece of tegmen with a long process.

Female genitalia. Unknown.

#### Type materials.

 **Holotype:** 1♂, **China, Sichuan:** Heizhugou Forest Park, Ebian, 29°2.51'N, 103°0.34'E , ca 1900m, 22.ix.2007, Wang XM leg. (SCAU). **Paratypes** (1): Yunnan: 1♂, Heiwadi Town, Gongshan, 27°46.48'N, 98°36.16'E , ca 2020m, 19.vii.2010, Wang XM leg. (SCAU).

#### Distribution.

China (Sichuan, Yunnan).

#### Etymology.

The specific epithet formed from the Latin prefixion *tri-* and noun *macula* referring to elytra with three spots.

### Serangium centrale

Wang & Ren sp. n.

urn:lsid:zoobank.org:act:A81A7070-E1BB-46EF-AEA0-E729F5C0D775

http://species-id.net/wiki/Serangium_centrale

Figs 6, 8 17 46–51 93 

#### Diagnosis.

This species is similar to *Serangium magnipunctatum* in general appearance, but it is easily distinguished from the latter by sharply pointed penis apex and extremely complex tegmen (Figs 37–39, 47–49). The male genitalia are similar to S. trimaculatum, but can be distinguished from latter by the shorter left paramere and the apex of penis guide (Figs 44–45, 48–49).

#### Description.

 TL: 2.08–2.27mm, TW: 1.88–1.98mm, TH: 1.09–1.15mm, TL/TW: 1.11–1.15; PL/PW: 0.51–0.52; EL/EW: 0.82–0.92.

Body minute, hemispherical, dorsum strongly convex, shiny and glabrous (Fig. 17). Head yellowish brown, pronotum reddish brown, scutellum dark brown. Elytra reddish brown, with a castaneous area in the middle of suture. Underside yellowish brown, legs yellowish brown.

Head transverse and ventrally flattened, 0.43× elytral width (HW/EW=1: 2.31); punctures on frons large, separated by 0.2–0.8 times their diameter, with sparse setae; eyes moderately large and coarsely faceted, widest interocular distance 0.54× head width. Antenna 9-segmented, terminal segment large, elongate oval and flat, apex angular (Fig. 6).

Pronotum short and strongly transverse, 0.70× elytral width (PW/EW=1: 1.43), densely covered in moderated large punctures associated with long dense setae, punctures slightly smaller than those on head, separated by 0.5–1.5 times their diameter. Punctures on elytra fine and sparse, smaller than those on pronotum, separated by 2.0–4.0 times their diameter, with a few large inside punctures at basal margins and two row of large inner punctures along elytral suture. Prosternum mat, with long dense setae. Mesoventrite small, transverse, surface mat weakly furrowed and impunctate. Metaventrite shiny and glabrous, basal half with inconspicuous median discrimen; punctures fine and sparse, separated by 2.0–4.0 times their diameter.

Male genitalia. Penis long and slender, strongly curved in whole length, apical half of siphon strongly narrowing apical, and apex very thin and sharply pointed, penis capsule broadening basally and without distinct inner and outer processes (Fig. 47). Tegmen strongly asymmetrical and extremely complicate (Figs 48–49). Penis guide relatively short with a distinctly pointed apex, a small gap at right side and a prominence at left side near the apex, bearing a tuft of setae (Fig. 49). Right lateral lobe of tegmen relatively short and wide, bearing a tuft of setae. Left lateral lobe of tegmen slightly longer and narrower than right, without any hairs (Fig. 49). Basal piece of tegmen with a long process.

Female genitalia. Genital plate elongate triangular, apical part of the plate narrow and parallel-sided with a rouned apex, stylus of the genital plate very elongate with a few long setae at its apex (Fig. 51). Spermatheca divided into two globular parts, each with a small pinch-like projection (Fig. 50).

#### Type materials.

 **Holotype**: 1♂, **China, Yunnan:** Dadugang, Puer, 22°22.35'N, 100°56.68'E , ca 950m, 26.iv.2008, Wang XM leg. (SCAU). **Paratypes** (2): 1♂1♀, same data as holotype (SCAU).

#### Distribution.

China (Yunnan).

#### Etymology.

The specific epithet formed from the Latin adjective *centrale* referring to elytra with a central dark spot.

### Serangium leigongicus

Wang & Ren sp. n.

urn:lsid:zoobank.org:act:0706CBF5-A39C-4A1E-8218-117C3D6D9889

http://species-id.net/wiki/Serangium_leigongicus

Figs 18 52–55 93 

#### Diagnosis.

This species is similar to *Serangium centrale* in male genitalia, but it is easily distinguished from the latter by minute body size, slender penis, shorter penis capsule, apex of penis guide with a triangular process at left side (Figs 47–49, 53–55).

#### Description.

 TL: 1.58mm, TW: 1.35mm, TH: 0.40mm, TL/TW: 1.17; PL/PW: 0.45; EL/EW: 0.93.

Body minute, hemispherical, dorsum strongly convex, shiny and glabrous (Fig. 18). Head brown, except frons yellowish brown. Pronotum and scutellum dark brown. Elytra burgundy, with a dark area in the middle of suture. Underside dark red. Legs yellowish brown.

Head transverse and ventrally flattened, 0.44× elytral width (HW/EW=1: 2.28); punctures on frons moderated large, separated by 0.5–1.5 times their diameter, with sparse setae; eyes moderately large and coarsely faceted, widest interocular distance 0.56× head width. Antenna 9-segmented, terminal segment large, elongate oval and flat, apex angular.

Pronotum short and strongly transverse, 0.76× elytral width (PW/EW=1: 1.32), sparsely covered in fine punctures associated with moderately dense setae, punctures smaller than as those on head, separated by 1.0–3.0 times their diameter. Punctures on elytra fine, similar as those on pronotum, separated by 2.0–4.0 times their diameter, with a row of evenly spaced setae along margin. Prosternum mat, shagreened and impunctate. Mesoventrite glabrous. Metaventrite shiny and glabrous, without median discrimen; punctures fine and sparse, separated by 2.0–5.0 times their diameter, slightly larger and denser in center.

Male genitalia. Penis long and slender, strongly curved in whole length, apical half of siphon strongly narrowing apical, and apex very thin and sharply pointed, penis capsule broadening basally and with distinct inner and outer processes (Fig. 53). Tegmen strongly asymmetrical and extremely complicate (Figs 54–55). Penis guide relatively short with a distinctly pointed apex, a small triangular process at left side and a prominence at left side near the apex, bearing dense setae dorsally and ventrally (Fig. 55). Right lateral lobe of tegmen short, bearing spaesrly setae. Left lateral lobe of tegmen slightly longer than right, without any setae (Fig. 55). Basal piece of tegmen with a long process.

Female genitalia. Unknown.

#### Type materials.

 **Holotype:** 1♂, **China, Guizhou:** Xiaodanjiang, Leigongshan National Natural Reserve, Leishan, 26°26.53'N, 108°15.45'E , ca 1160m, 12.x.2008, Liang JB leg. (SCAU).

#### Distribution.

China (Guizhou).

#### Etymology.

The specific epithet is named after Leigongshan, the type locality of this ladybird.

### Serangium latilobum

Wang & Ren sp. n.

urn:lsid:zoobank.org:act:0C2D2BE9-06F2-4DFC-AAAB-A29B8156A700

http://species-id.net/wiki/Serangium_latilobum

Figs 19 56–61 94 

#### Diagnosis.

**Figures 19–24.**
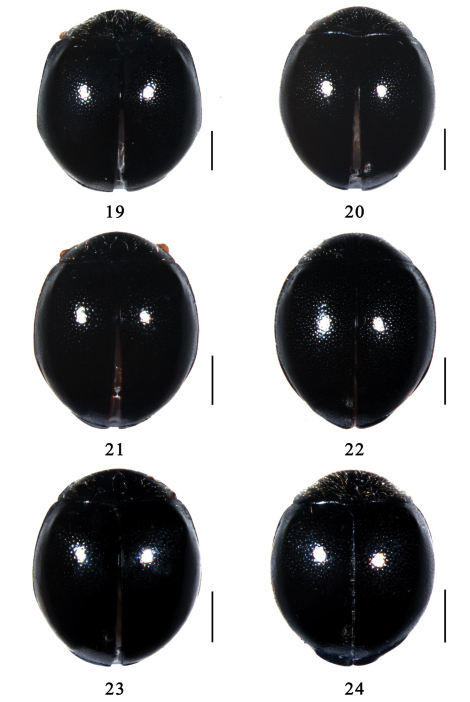
Dorsal view.**19** *Serangium latilobum*Wang & Ren, sp. n. **20** *Serangium digitiforme* Wang & Ren, sp. n. **21** *Serangium drepnicum* Xiao **22** *Serangium punctum* Miyatake **23** *Serangium dulongjiang* Wang, Ren & Chen, sp. n.; **24** *Serangium contortum* Wang & Ren, sp. n. Scale bars: 0.5mm.

This species is similar to *Serangium japonicum* in general appearance, but it can be distinguished from the latter by lager body size, uniform black pronotum without other color and elytra with very conspicuous swelling (Fig. 19). The male genitalia are also diagnostic: penis is moderated stout (Fig. 57), right paramere is indistinct (Fig. 58), penis guide is wide at basal half, sharply narrowed at middle with a finger-shape apex (Fig. 59).

#### Description.

 TL: 2.14–2.18mm, TW: 1.91–1.96mm, TH: 0.99–1.15mm, TL/TW: 1.11–1.12; PL/PW: 0.49–0.53; EL/EW: 0.87–0.88.

Body minute, hemispherical, dorsum strongly convex, shiny and glabrous (Fig. 19). Dorsum uniformly black. Head yellow. Underside black, except prosternum orange. Legs yellowish brown.

Head transverse and ventrally flattened, 0.44× elytral width (HW/EW=1: 2.29); punctures on frons fine, separated by 0.5–1.5 times their diameter, with sparse setae; eyes moderately large and coarsely faceted, widest interocular distance 0.52× head width. Antenna 9-segmented, terminal segment large, elongate oval and flat, apex angular.

Pronotum short and strongly transverse, 0.70× elytral width (PW/EW=1: 1.43), covered in fine punctures associated with long dense setae, punctures similar as those on head, separated by 1.0–2.0 times their diameter. Punctures on elytra fine and sparse, similar to those on pronotum, separated by 2.0–3.0 times their diameter, with a few long setae at humeral angles and a row of evenly spaced setae along margin. Prosternum mat, with sparse setae. Mesoventrite glabrous and impunctate, with sparse setae. Metaventrite shiny and glabrous, basal half with distinctly median discrimen; punctures around median discrimen large and dense, with short thick setae, punctures on the rest parts indistinct, with sparse setae.

Male genitalia. Penis strongly curved in whole length, with a small prominence at 1/5, apex slightly curved, penis capsule broadening basally and with indistinct inner and outer processes (Fig. 57). Tegmen rather slender and strongly asymmetrical (Figs 58–59). Penis guide in ventral view wide at basal half, sharply narrowed at middle, then contort, forming a distinct gap in middle, apical half elongate finger-shape. Left paramere in ventral view indistinct, bearing a few long setae, and right piece very short, bearing a few long setae (Fig. 59). Penis guide in lateral view widest at base, gradually tapering to apex, basal 1/5 with a small angular prominence, apex sharply pointed (Fig. 58).

Female genitalia. Genital plate triangular with a rounded apex, slightly concaved in middle, sparsely hairy on the apical portion, stylus rather long, bearing few setae (Fig. 61). Spermatheca divided into two parts, one of which is somewhat globular with a constriction and two small pinch-like projections, the other is short tubular, becoming slightly more slender distally (Fig. 60).

**Figures 25–35.**
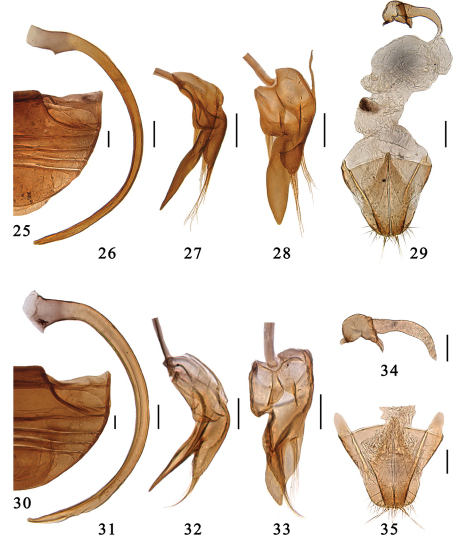
**25–29.** *Serangium japonicum* Chapin. **25** abdomen **26–28** male genitalia: **26** penis **27** tegmen, lateral view **28** tegmen, ventral view **29** female genitalia. **30–35.** *Serangium clauseni*(Chapin). **30** abdomen **31–33** male genitalia: **31** penis **32** tegmen, lateral view **33** tegmen, ventral view. **34–35** female genitalia: **34** spermatheca **35** ovipositor. Scale bars: 0.1mm.

**Figures 36–45.**
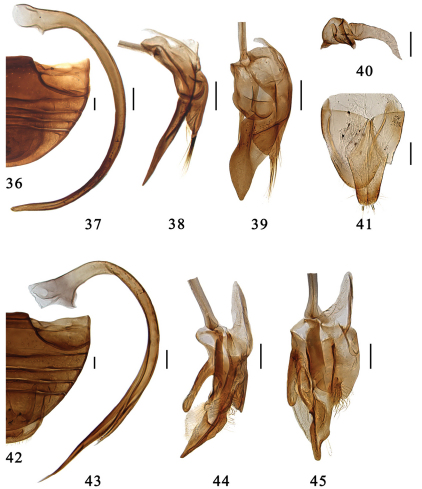
**36–41.** *Serangium magnipunctatum* Wang & Ren, sp. n. **36** abdomen. **37–39** male genitalia: **37** penis **38** tegmen, lateral view **39** tegmen, ventral view. **40–41** female genitalia: **40** spermatheca **41** ovipositor. **42–45.** *Serangium trimaculatum*Wang & Ren, sp. n. **42** abdomen. **43–45** male genitalia: **43** penis **44** tegmen, lateral view **45** tegmen, ventral view. Scale bars: 0.1mm.

**Figures 46–55.**
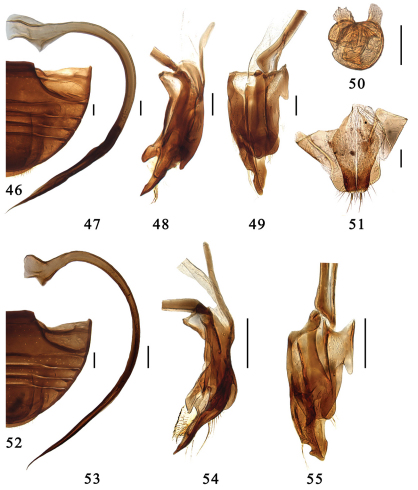
**46–51.** *Serangium centrale*Wang & Ren, sp. n. **46** abdomen. **47–49** male genitalia: **47** penis **48** tegmen, lateral view **49** tegmen, ventral view. **50–51** female genitalia: **50** spermatheca **51** ovipositor. **52–55.** *Serangium leigongicus* Wang & Ren, sp. n. **52** abdomen. **53–55** male genitalia: **53** penis **54** tegmen, lateral view **55** tegmen, ventral view. Scale bars: 0.1mm.

#### Type materials.

 **Holotype**: 1♂, **China, Yunnan:** Heiwadi Town, Gongshan, [27°46.48'N, 98°36.16'E ], ca 2020m, 19.vii.2010, Wang XM leg. (SCAU). **Paratypes** (28): **Yunnan:** 10♂♂17♀♀, same data as holotype (6♂♂10♀♀, SCAU; 4♂♂7♀♀, IOZ); 1♂, Gaoligongshan National Natural Reserve, Baoshan, [24°59.92'N, 99°5.03'E ], ca 2000m, 19.ix.2006, Wang XM leg. (SCAU).

#### Distribution.

China (Yunnan).

#### Etymology.

The specific epithet formed from the Latin adjective *latilobus* referring to penis guide which is wide at basal half, sharply narrowed and forming a distinct gap in middle.

### Serangium digitiforme

Wang & Ren sp. n.

urn:lsid:zoobank.org:act:60D8CEB6-9E49-4BA8-983F-811F1C3FF2A0

http://species-id.net/wiki/Serangium_digitiforme

Figs 20 62–67 94 

#### Diagnosis.

This species is very similar to *Serangium  latilobum* in general appearance and male genitalia, but it is distinguished from the latter by penis distinctly broadening at apical 1/5–2/5, penis guide widest at base, gradually narrowing to middle (Fig. 65). In *Serangium latilobum*, penis only has a small pominence at 1/5 (Fig. 57), penis guide wide at basal half, sharply narrowed in middle (Fig. 59).

#### Description.

 TL: 2.14–2.21mm, TW: 1.80–1.85mm, TH: 1.01–1.05mm, TL/TW: 1.19–1.20; PL/PW: 0.46–0.48; EL/EW: 0.95–1.00.

Body minute, hemispherical, dorsum strongly convex, shiny and glabrous (Fig. 20). Dorsum uniformly black. Head dark brown. Underside dark brown. Legs dark red, except tarsi yellow.

Head transverse and ventrally flattened, 0.39× elytral width (HW/EW=1: 2.60); punctures on frons fine, separated by 1.0–3.0 times their diameter, with sparse setae; eyes moderately large and coarsely faceted, widest interocular distance 0.52× head width. Antenna 9-segmented, terminal segment large, elongate oval and flat, apex angular.

Pronotum short and strongly transverse, 0.70× elytral width (PW/EW=1: 1.43), covered in fine and dense punctures associated with moderately dense setae, similar to those on head, separated by 0.5–1.5 times their diameter. Punctures on elytra fine and sparse, slightly smaller than those on pronotum, separated by 2.0–3.0 times their diameter, with a few long setae at humeral angles and a row of evenly spaced setae along margin. Prosternum mat and shagreened, with sparse setae. Mesoventrite glabrous and impunctate, with sparse setae. Metaventrite shiny and glabrous, basal half with distinctly median discrimen; punctures around median discrimen very large and dense, with short thick setae, punctures on the rest parts moderated and sparse, separated by 2.0–4.0 times their diameter, with sparse setae.

Male genitalia. Penis strongly curved in whole length, distinct distinctly broadening at 1/5–2/5, apex narrowing and slightly curved, penis capsule broadening basally and with indistinct inner and outer processes (Fig. 63). Tegmen rather slender and strongly asymmetrical (Figs 64–65). Penis guide in ventral view widest at base, strongly narrowing to middle, then contorted, apical half finger-shape. Left and right paramere in ventral view short, bearing a few long setae (Fig. 65). Penis guide in lateral view widest at base, gradually tapering to apex (Fig. 64).

Female genitalia. Genital plate triangular with a rounded apex, slightly concaved in middle, sparsely hairy on the apical portion, stylus rather long, bearing few setae (Fig. 66). Spermatheca divided into two parts, one of which is somewhat globular with a constriction and two small pinch-like projections, the other is short tubular, becoming slightly more slender distally (Fig. 65).

**Figures 56–67.**
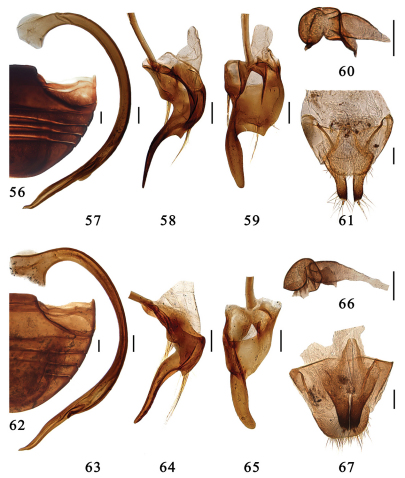
**56–61.** *Serangium latilobum*Wang & Ren, sp. n. **56** abdomen. **57–59** male genitalia: **57** penis **58** tegmen, lateral view **59** tegmen, ventral view. **60–61** female genitalia: **60** spermatheca **61** ovipositor. **62–67.** *Serangium digitiforme* Wang & Ren, sp. n. **62** abdomen. **63–65** male genitalia: **63** penis **64** tegmen, lateral view. **65** tegmen, ventral view. **66–67** female genitalia: **66** spermatheca **67** ovipositor. Scale bars: 0.1mm.

#### Type materials.

 **Holotype:** 1♂, **China, Hubei:** Hongping, Shennongjia National Natural Reserve, 31°40.07'N, 110°25.73'E , ca 1830m, 19.vii.1997, Peng ZQ leg. (SCAU). **Paratypes** (6): **Hubei:** 1♂, Liujiawu, Shennongjia National Natural Reserve, 31°33.14'N, 110°21.56'E , ca 1790m, 10.vii.1989, Ren SX leg. (SCAU); **Sichuan:** 1♂, Liziping, Shimian, 28°59.75N, 102°18.04'E , ca 2000m, 26.ix.2007, Wang XM leg. (SCAU); **Guizhou:** 1♂, Lianhuaping, Leigongshan Mountain, Leishan, 26°21.89'N, 108°9.17'E , ca 1240m, 8.x.2008, Liang JB leg. (SCAU); 1♂, Maoping, Leigongshan Mountain, Leishan, 26°22.48'N, 108°9.85'E , ca 1410m, 8.x.2008, Liang JB leg. (SCAU); **Gansu:** 1♂1♀, Maijishan, Tianshui, 34°20.79'N, 106°0.68'E , ca 1500m, 10.viii.2008, Wang XM leg. (SCAU).

#### Distribution.

China (Gansu, Guizhou, Hubei, Sichuan).

#### Etymology.

The specific epithet formed from the Latin noun *digitus* and *forma*, referring to digitiform penis guide.

### Serangium drepnicum

Xiao, 1992

http://species-id.net/wiki/Serangium_drepnicum

Figs 21 68–73 94

Serangium drepnicum : Xiao & Li, 1992: 368; Ślipiński & Burckhardt, 2006: 50; Ren et al., 2009: 40.
Serangium lygacum : Pang et al., 2004: 66.

#### Diagnosis.

This species is close to *Serangium japonicum* in general appearance and genitalia, but can be distinguished as follows by indistinctly right paramere and flat and wide penis guide (Figs 70–71).

#### Description.

 TL: 1.98–2.11mm, TW: 1.68–1.81mm, TH: 0.96–0.99mm, TL/TW: 1.16–1.18; PL/PW: 0.47–0.48; EL/EW: 0.94–0.96.

Body minute, hemispherical, dorsum strongly convex, shiny and glabrous (Fig. 21). Dorsum uniformly black. Head yellow, with basal frons black. Underside black, except prosternum orange. Legs reddish brown, except tibiae and tarsi yellow.

Head transverse and ventrally flattened, 0.41× elytral width (HW/EW=1: 2.43); punctures on frons fine, separated by 1.0–4.0 times their diameter, with sparse setae; eyes moderately large and coarsely faceted, widest interocular distance 0.48× head width. Antenna 9-segmented, terminal segment large, elongate oval and flat, apex angular.

Pronotum short and strongly transverse, 0.70× elytral width (PW/EW=1: 1.43), covered in fine punctures associated with long sparse setae, separated by 1.0–3.0 times their diameter. Punctures on elytra fine and sparse, smaller than those on pronotum, separated by 2.0–4.0 times their diameter, with a row of evenly spaced setae along margin. Prosternum mat, with sparse setae. Mesoventrite glabrous and impunctate, with sparse setae. Metaventrite shiny and glabrous, basal half with distinctly median discrimen; punctures fine and sparse, separated by 2.0–5.0 times their diameter, with short sparse setae.

Male genitalia. Penis strongly curved in whole length, apex shortly narrowing and rounded, penis capsule indistinct (Fig. 69). Tegmen rather slender and strongly asymmetrical (Figs 70–71). Penis guide in ventral view flattened and elongate tongue-shape. Left paramere in ventral view indistinct bearing a few long setae, and right piece distinctly projecting, bearing a few long setae (Fig. 71). Penis guide in lateral view long and thin, straight, apex sharply pointed (Fig. 70).

Female genitalia. Genital plate elongate triangular with a rounded apex, sparsely hairy on the apical portion, stylus long, bearing few setae (Fig. 73). Spermatheca divided into two parts, one of which is somewhat globular with a feeble constriction and two small pinch-like projections, the other is long tubular, becoming slightly more slender distally (Fig. 72).

**Figures 68–79.**
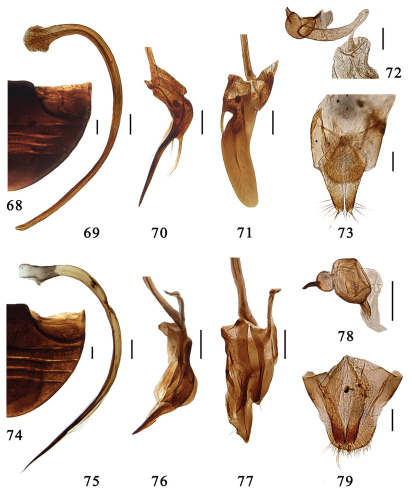
**68–73.** *Serangium drepnicum* Xiao. **68** abdomen; **69–71** male genitalia: **69** penis **70** tegmen, lateral view **71** tegmen, ventral view. **72–73** female genitalia: **72** spermatheca **73** ovipositor. **74–79.** *Serangium punctum* Miyatake **74** abdomen. **75–77** male genitalia: **75** penis **76** tegmen, lateral view **77** tegmen, ventral view. **78–79** female genitalia: **78** spermatheca **79** ovipositor. Scale bars: 0.1mm.

#### Types.

 **Holotype:** 1♂, Hefeng, Hubei, 29°53.0'N, 110°2.38'E , ca 800m, 27.vii.1989, Xiao NN leg. (KIZ); **Paratypes:** 2♂♂, Lichuan, Hubei, 30°19.12'N, 108°55.35'E , ca 800m, 2.viii.1989, Xiao NN leg. (KIZ).

#### Other specimens examined.

 **China, Fujian:** 1♂1♀, Sangang, Wuyi, 26°11.89'N, 119°15.97'E , ca 380m, 16.viii.1984, Pang XF leg.; **Henan:** 2♂♂1♀, Baiyushan, Songxian, 33°40.14'N, 110°50.56'E , ca 1520m, 1375m, 14.vii.2009, Wang XM et al. Leg.; 1♂, Baotianman, Neixiang, 33°32.15'N, 111°51.38'E , ca 1030m, 6.vii.2009, Wang XM Leg.

#### Distribution.

China (Fujian, Henan, Hubei).

### Serangium punctum

(Miyatake, 1963)

http://species-id.net/wiki/Serangium_punctum

Figs 22 74–79 95

Serangium punctum : Miyatake, 1963: 13; Sasaji, 1971: 57.
Serangium ezoense : Miyatake, 1963: 14.

#### Diagnosis.

This species is easily recognized by frons, meso- and metaventrite with large dense punctures and the special construction of penis guide (Figs 75–77).

#### Description.

 TL: 2.08mm, TW: 1.85mm, TH: 0.92mm, TL/TW: 1.13; PL/PW: 0.45; EL/EW: 0.86.

Body minute, hemispherical, dorsum strongly convex, shiny and glabrous (Fig. 22). Dorsum uniformly black. Head black. Underside black. Legs dark brown, except tibiae and tarsi yellow.

Head transverse and ventrally flattened, 0.38× elytral width (HW/EW=1: 2.67); punctures on frons large and dense, separated by 0.5–1.0 times their diameter, with sparse setae; eyes moderately large and coarsely faceted, widest interocular distance 0.52× head width. Antenna 9-segmented, terminal segment large, elongate oval and flat, apex angular.

Pronotum short and strongly transverse, 0.71× elytral width (PW/EW=1: 1.40), evenly covered in moderately large punctures associated with long sparse setae, punctures slightly smaller than those on head, separated by 1.5–3.0 times their diameter. Punctures on elytra fine, slightly smaller than those on pronotum, separated by 2.0–4.0 times their diameter, with a few long setae at humeral angles and a row of evenly spaced setae along margin. Prosternum mat and shagreened. Meso- and metaventrites without distinctly median discrimen; punctures large and sparse, separated by 1.5–3.0 times their diameter, with sparse setae.

Male genitalia. Penis long and slender, strongly curved in whole length, apical half of siphon strongly narrowing apical, and apex very thin and sharply pointed, penis capsule broadening basally and without distinct inner and outer processes (Fig. 75). Tegmen strongly asymmetrical and extremely complicate (Figs 76–77). Penis guide moderatedly elongate, with a pointed apex, a small triangular process at left side near the apex and an angulate prominence at middle of left side, bearing sparse setae (Fig. 77). Right paramere relatively long, 1/2 of penis guide length, bearing minute setae. Left lateral lobe of tegmen slightly longer than right, flat and elongate oval without any hairs (Fig. 77). Paramere in lateral view arcuate, with a sharply pointed apex (Fig. 76). Basal piece of tegmen with a long process.

Female genitalia. Genital plate elongate triangular, apical part of the plate narrow and parallel-sided with a rouned apex, stylus of the genital plate very elongate with a few long setae at its apex (Fig. 79). Spermatheca divided into two parts, one of which is globular with a small pinch-like projection, the other nearly hemispherical, with a small pinch-like projection (Fig. 78).

#### Specimens examined:

**Gansu:** 1♂, Maiji, Tianshui, 34°20.79'N, 106°0.28'E , ca 1650m, 6.viii.2009, Wang XM leg. **Guizhou:** 1♀, Xiaodanjiang, Leigongshan National Natural Reserve, Leishan, 26°26.53'N, 108°15.45'E , ca 1160m, 12.x.2008, Liang JB leg.

#### Distribution.

China (Gansu, Guizhou) **new distribution**; Japan

### Serangium dulongjiang

Wang, Ren & Chen sp. n.

urn:lsid:zoobank.org:act:B8DCED81-ACCD-4F34-9B09-AC6288CD2D87

http://species-id.net/wiki/Serangium_dulongjiang

Figs 23 80–85 95 

#### Diagnosis.

This species is close to *Serangium digitiforme*in general appearance and male genitalia, but it is distinguished from the latter as follow: penis is long and slender (Fig. 81), penis guide is elongated tongue-shaped (Fig. 83), and the first part of spermatheca has a strong constriction in middle (Fig. 84). In *Serangium digitiforme*, penis is moderately long and stout, distinctly broadening at apical 1/5–2/5 (Fig. 63), penis guide is finger-shaped (Fig. 65), and the first part of spermatheca has a feebly constriction in middle (Fig. 66).

**Figures 80–91.**
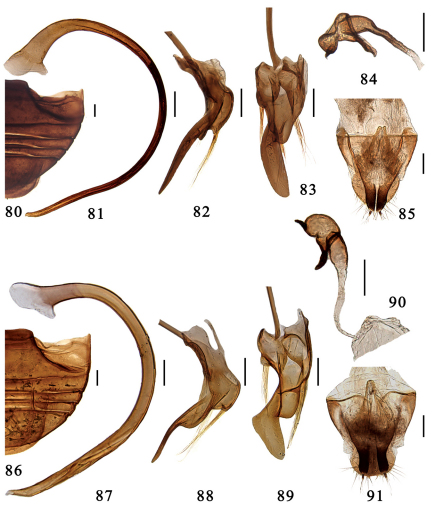
**80–85.** *Serangium dulongjiang* Wang, Ren & Chen, sp. n. **80** abdomen. **81–83** male genitalia: **81** penis **82** tegmen, lateral view **83** tegmen, ventral view. **84–85** female genitalia: **84** spermatheca **85** ovipositor. **86–91.** *Serangium contortum* Wang & Ren, sp. n. **86** abdomen. **87–89** male genitalia: **87** penis **88** tegmen, lateral view **89** tegmen, ventral view. **90–91** female genitalia: **90** spermatheca **91** ovipositor. Scale bars: 0.1mm.

#### Description.

 TL: 2.01–2.14mm, TW: 1.68–1.81mm, TH: 0.86–0.96mm, TL/TW: 1.18–1.20; PL/PW: 0.44–0.49; EL/EW: 0.96–1.00.

Body minute, hemispherical, dorsum strongly convex, shiny and glabrous (Fig. 23). Dorsum uniformly black. Head orange to dark brown. Underside black. Legs yellowish brown to dark red, except tarsi yellow.

Head transverse and ventrally flattened, 0.41× elytral width (HW/EW=1: 2.43); punctures on frons fine, separated by 1.5–2.5 times their diameter, with sparse setae; eyes moderately large and coarsely faceted, widest interocular distance 0.52× head width. Antenna 9-segmented, terminal segment large, elongate oval and flat, apex angular.

Pronotum short and strongly transverse, 0.71× elytral width (PW/EW=1: 1.42), densely covered in fine punctures associated with long dense setae, punctures similar to those on head, separated by 0.5–1.0 times their diameter. Punctures on elytra fine and sparse, similar to those on pronotum, separated by 2.0–3.0 times their diameter, with a row of evenly spaced setae along margin. Prosternum mat, with sparse setae. Mesoventrite glabrous and impunctate. Metaventrite shiny and glabrous, without distinctly median discrimen; punctures in center large and dense, with short thick setae.

Male genitalia. Penis very long and slender, strongly curved in whole length, apex shortly narrowing and rounded, penis capsule indistinct (Fig. 81). Tegmen rather slender and strongly asymmetrical (Figs 82–83). Penis guide in ventral view widest at base, gradually narrowing to middle, then strongly narrowed and contorted, apical elongate tongue-shape. Left paramere in ventral view short bearing a few long setae, and right piece short but distinctly projecting, bearing a few long setae (Fig. 83). Penis guide in lateral view long and thin, almost straight, with a angular prominence near the base, apex pointed (Fig. 82).

Female genitalia. Genital plate elongate triangular with a rounded apex, slightly concaved in middle of outer margin, apical portion with sparsely setae, stylus long, bearing few setae (Fig. 85). Spermatheca divided into two parts, one of which is somewhat globular with a strong constriction and two pinch-like projections, the other is tubular, becoming slightly more slender distally (Fig. 84).

#### Type materials.

 **Holotype:** 1♂, **China, Yunnan:** Longyuan Village, Dulongjiang, Gongshan, 28°1.16'N, 98°18.88'E , ca 1500m, 26.vii.2010, Wang XM leg. (SCAU). **Paratypes** (46): **Yunnan:** 7♂♂22♀♀, same data as holotype (5♂♂15♀♀, SCAU; 2♂♂7♀♀, IOZ); 1♂2♀♀, Bapo Village, Dulongjiang, Gongshan, 27°43.84'N, 98°20.71'E , ca 1390m, 1400m, 28.vii.2010, Wang XM leg. (SCAU); 1♀, Maku Village, Dulongjiang, Gongshan, 27°41.11'N, 98°18.19'E , ca 1400m, 28.vii.2010, Wang XM leg. (SCAU); 8♂♂5♀♀, Lushui, Pianma, 26°0.42'N, 98°39.46'E , ca 2300m, 10.viii.2010, Wang XM et al. leg. (5♂♂2♀♀, SCAU; 3♂♂3♀♀, IOZ).

#### Distribution.

China (Yunnan).

#### Etymology.

The specific epithet is named after Dulongjiang, the type locality of this ladybird.

### Serangium contortum

Wang & Ren sp. n.

urn:lsid:zoobank.org:act:FCD37526-FC0C-4FA2-86D0-38183E3653D0

http://species-id.net/wiki/Serangium_contortum

Figs 24 86–91 95 

#### Diagnosis.

This species is easily recognized by its metaventrite without median discrimen and the contorted penis guide (Figs 88–89).

#### Description.

 TL: 1.62–1.78mm, TW: 1.42–1.58mm, TH: 0.69–0.96mm, TL/TW: 1.13–1.14; PL/PW: 0.48–0.49; EL/EW: 0.83–0.91.

Body minute, hemispherical, dorsum strongly convex, shiny and glabrous (Fig. 24). Dorsum uniformly black. Head yellow. Underside reddish brown, except meso- and metaventrite black. Legs yellowish brown, except femora reddish brown.

Head transverse and ventrally flattened, 0.42× elytral width (HW/EW=1: 2.40); punctures on frons moderately large, separated by 0.5–1.5 times their diameter, with sparse setae; eyes moderately large and coarsely faceted, widest interocular distance 0.53× head width. Antenna 9-segmented, terminal segment large, elongate oval and flat, apex angular.

Pronotum short and strongly transverse, 0.72× elytral width (PW/EW=1: 1.39), covered in fine inconspicuous punctures associated with long dense setae, punctures slightly smaller than those on head, separated by 1.0–1.5 times their diameter. Punctures on elytra fine and sparse, smaller than those on pronotum, separated by 2.0–3.0 times their diameter, with a row of evenly spaced setae along margin. Prosternum mat, punctures fine and inconspicuous, with sparse setae. Mesoventrite glabrous. Metaventrite shiny and glabrous, without distinctly b median discrimen; 5–6 large and dense punctures forming two cluster in the center, with short thick setae, punctures on the rest parts indistinct, with sparse setae.

Male genitalia. Penis long and stout, strongly curved in whole length, apex strongly narrowing and rounded, penis capsule indistinct (Fig. 87). Tegmen rather slender and strongly asymmetrical (Figs 88–89). Penis guide in ventral view strongly contorted in middle. Left paramere in ventral view short bearing a few long setae, and right piece short but distinctly projecting, bearing a few long setae (Fig. 89). Penis guide in lateral view wide in basal half, then sharply narrowed, apical half very thin, apex pointed (Fig. 88).

Female genitalia. Genital plate elongate triangular with a rounded apex, distinctly concaved in middle of outer margin, apical portion with sparsely setae, stylus long, bearing few setae (Fig. 91). Spermatheca divided into two parts, one of which is somewhat globular with a feeble constriction and two pinch-like projections, the other is tubular, becoming slightly more slender distally (Fig. 90).

#### Type materials.

 **Holotype:** 1♂, **China, Yunnan:** Heiwadi Town, Gongshan, 27°46.48'N, 98°36.16'E , ca 2020m, 19.vii.2010, Wang XM leg. (SCAU). **Paratypes** (10): **Yunnan:** 4♂♂4♀♀, same data as holotype (2♂♂2♀♀, SCAU; 2♂♂2♀♀, IOZ); **Guangxi:** 1♂, Jiuniutang, Maoershan National Natural Reserve, Guilin, 25°50.55'N, 110°22.85'E , ca 1390m, 18.x.2004, Wang XM leg. (SCAU); **Hubei:** 1♂, Wudangshan, Shiyan, 32°24.50'N, 111°1.31'E , ca 1090m, 17.vii.1997, Peng ZQ leg. (SCAU).

#### Distribution.

China (Guangxi, Hubei, Yunnan).

#### Etymology.

The specific epithet formed from the Latin adjective *contortus*, referring to contorted penis guide of tegmen.

**Figure 92.**
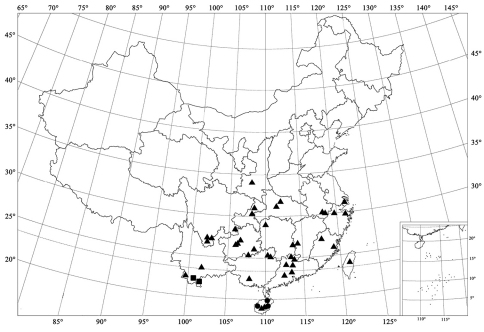
Distribution map. *Serangium japonicum* Chapin (▲); *Serangium clauseni* (Chapin) (●); *Serangium magnipunctatum* Wang & Ren, sp. n. (■).

**Figure 93.**
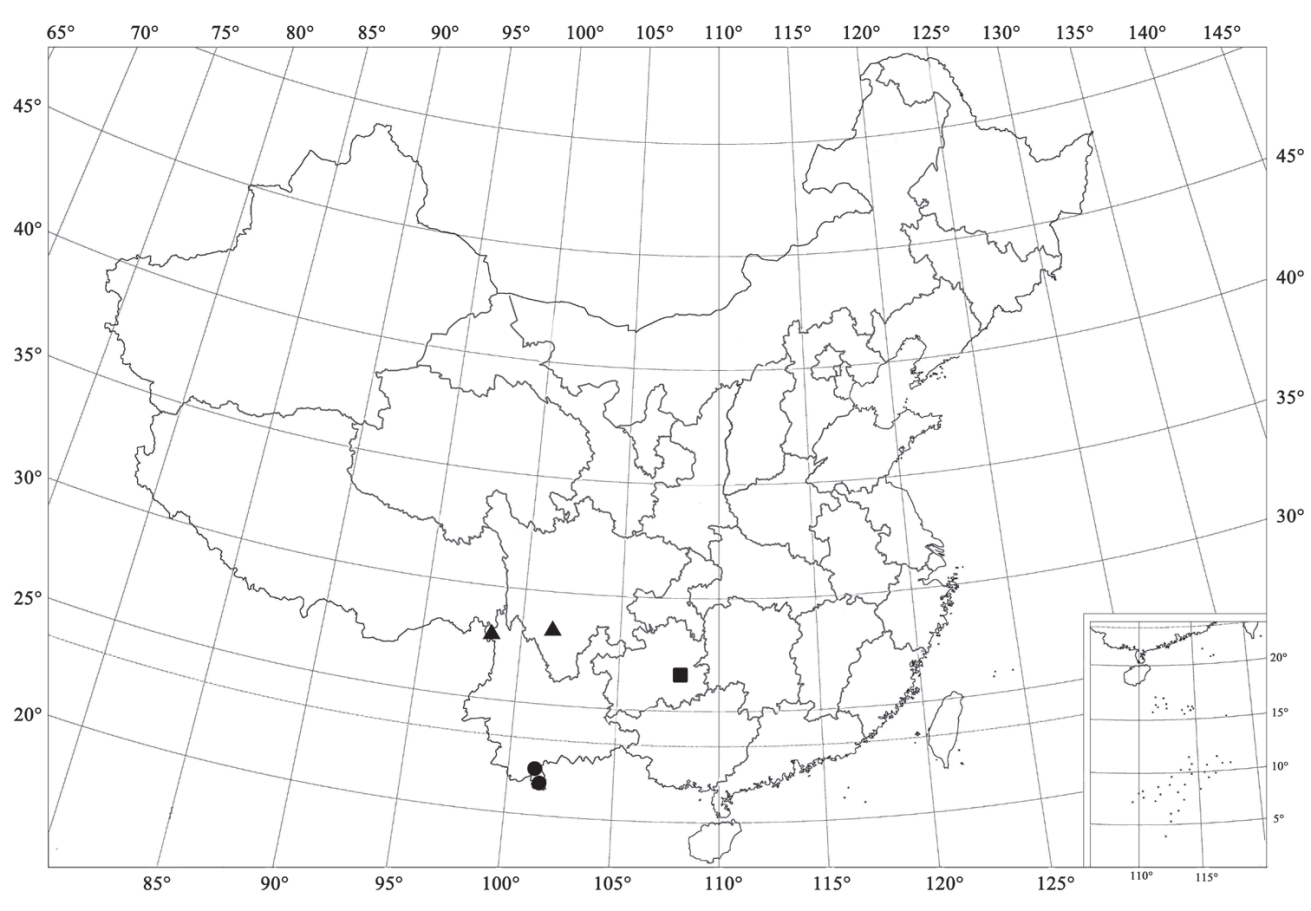
Distribution map. *Serangium trimaculatum*Wang & Ren, sp. n. (▲); *Serangium centrale* Wang & Ren, sp. n. (●); *Serangium leigongicus* Wang & Ren, sp. n. (■).

**Figure 94.**
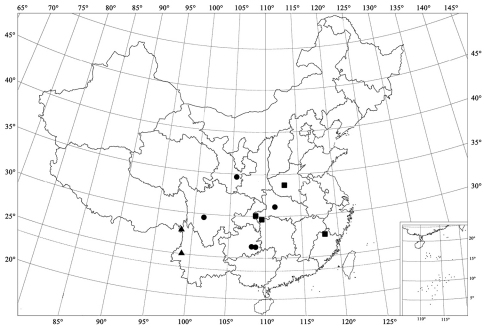
Distribution map. *Serangium latilobum*Wang & Ren, sp. n. (▲); *Serangium digitiforme* Wang & Ren, sp. n. (●); *Serangium drepnicum* Xiao (■).

**Figure 95.**
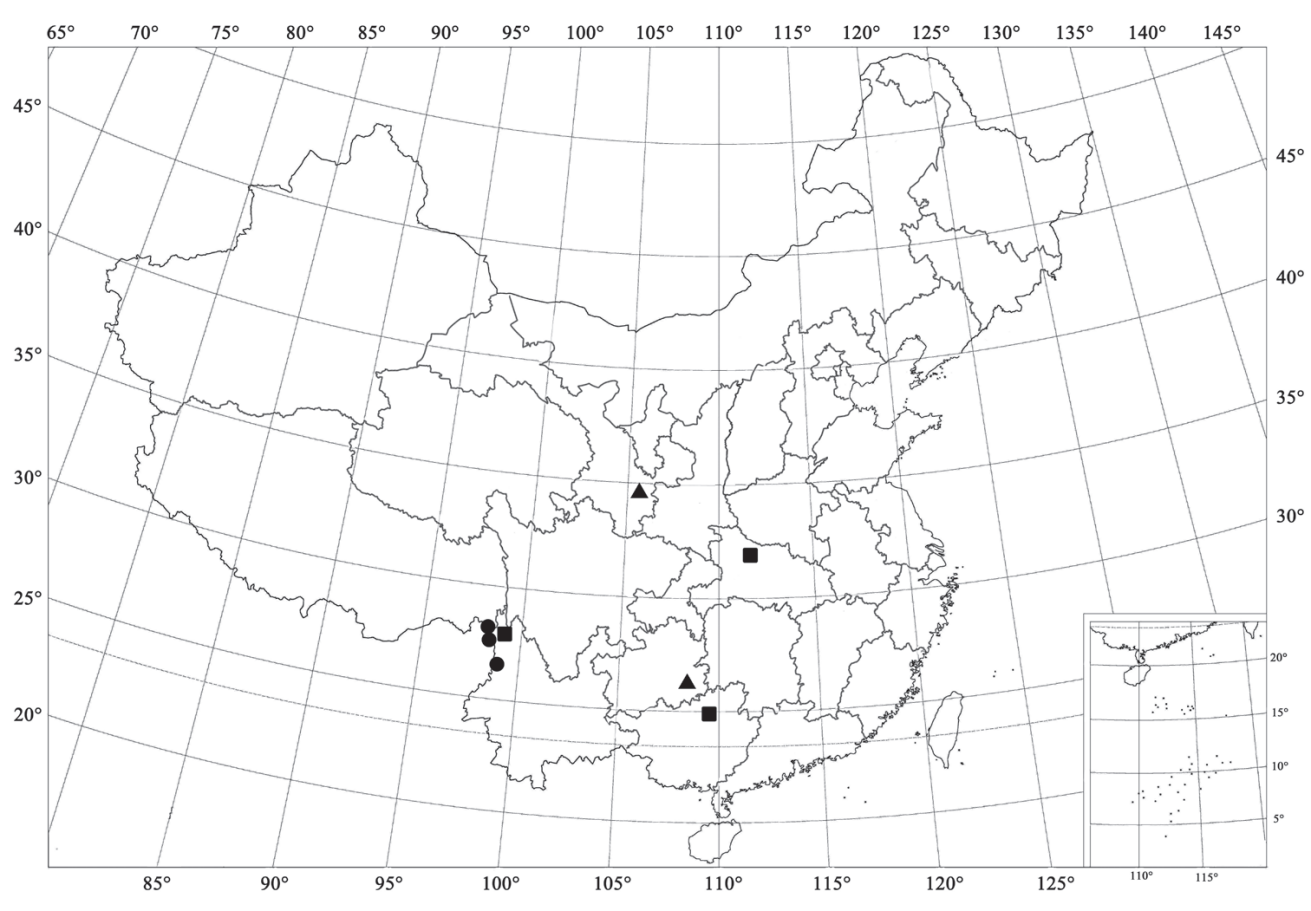
Distribution map. *Serangium punctum* Miyatake (▲); *Serangium dulongjiang* Wang, Ren & Chen, sp. n. (●); *Serangium contortum* Wang & Ren, sp. n. (■).

## Supplementary Material

XML Treatment for Serangium

XML Treatment for Serangium japonicum

XML Treatment for Serangium clauseni

XML Treatment for Serangium magnipunctatum

XML Treatment for Serangium trimaculatum

XML Treatment for Serangium centrale

XML Treatment for Serangium leigongicus

XML Treatment for Serangium latilobum

XML Treatment for Serangium digitiforme

XML Treatment for Serangium drepnicum

XML Treatment for Serangium punctum

XML Treatment for Serangium dulongjiang

XML Treatment for Serangium contortum
